# Fish-oil esters of plant sterols differ from vegetable-oil sterol esters in triglycerides lowering, carotenoid bioavailability and impact on plasminogen activator inhibitor-1 (PAI-1) concentrations in hypercholesterolemic subjects

**DOI:** 10.1186/1476-511X-6-28

**Published:** 2007-10-25

**Authors:** Peter JH Jones, Isabelle Demonty, Yen-Ming Chan, Yael Herzog, Dori Pelled

**Affiliations:** 1 School of Dietetics and Human Nutrition, McGill University, Sainte-Anne-de-Bellevue, Canada; 2 R&D Department, Enzymotec LTD, Migdal HaEmeq, Israel

## Abstract

**Background:**

Consumption of plant sterol (PS) esters lower low-density lipoprotein (LDL)-cholesterol levels by suppressing intestinal absorption of cholesterol. Commercially available PS are mainly esterified to omega-6 fatty acid (FA), such as sunflower oil (SO) FA. Emerging trends include using other sources such as olive oil (OO) or omega-3 FA from fish oil (FO), known to exert potent hypotriglyceridemic effects. Our objective was to compare the actions of different FA esterified to PS on blood lipids, carotenoid bioavailability as well as inflammatory and coagulation markers.

**Methods:**

Twenty-one moderately overweight, hypercholesterolemic subjects consumed experimental isoenergetic diets enriched with OO (70% of fat), each lasting 28-day and separated by 4-week washout periods, using a randomized crossover design. Diets were supplemented with three PS esters preparations, PS-FO, PS-SO, or PS-OO. All PS treatments contained an equivalent of 1.7 PS g/d, and the PS-FO provided a total of 5.4 g/d FO FA (eicosapentaenoic and docosahexaenoic acids).

**Results:**

There were no differences between PS-containing diet effects on total cholesterol, LDL-cholesterol, or high-density lipoprotein (HDL)-cholesterol levels. However, PS-FO consumption resulted in markedly lower (P < 0.0001) fasting and postprandial triglyceride concentrations compared with PS-SO and PS-OO. These treatments affected plasma β-carotene (P = 0.0169) and retinol (P = 0.0244), but not tocopherol (P = 0.2108) concentrations. Consumption of PS-FO resulted in higher β-carotene (P = 0.0139) and retinol (P = 0.0425) levels than PS-SO and PS-OO, respectively. Plasma TNF-α, IL-6, C-reactive protein, prostate specific antigen, and fibrinogen concentrations were unaffected by the PS-interventions. In contrast, plasminogen activator inhibitor 1 (PAI-1) concentrations were lower (P = 0.0282) in the PS-FO-fed than the PS-SO, but not the PS-OO (P = 0.7487) groups.

**Conclusion:**

Our findings suggest that, in hypercholesterolemic subjects consuming an OO-based diet, PS-FO results in lowered blood triglyceride and PAI-1 concentrations, and higher fat-soluble vitamin levels in comparison to the vegetable oil FA esters of PS (PS-SO and PS-OO). Thus, PS-FO may offer hyperlipidemic subjects a more comprehensive lipid lowering approach while reducing the potential risk of decreased plasma carotenoid concentrations.

## Background

Complications of atherosclerosis are considered the leading cause of death in Western societies and have an extremely high incidence in type 2 diabetes mellitus patients [1]. It is well established that elevated serum low-density lipoprotein (LDL)-cholesterol concentrations are a major risk factor for atherosclerotic disease [2]. Recent reports favored LDL-cholesterol lowering not only in individuals with coronary artery disease or severely elevated blood cholesterol concentrations, but also in healthy persons with only mild-to-moderate hypercholesterolemia [3-5]. Plant sterols (PS) or phytosterols such as β-sitosterol, campesterol, and stigmasterol occur naturally and are structurally similar to cholesterol, but an additional hydrocarbon chain at carbon 24 and a double bond on the side chain [6]. PS intake in Western diets is estimated to be equivalent to that of dietary cholesterol, i.e. below 500 mg/d. Recently, consumption of 2 g/d of PS for LDL-cholesterol lowering was recommended by the National Cholesterol Education Program [3] as part of the standard therapy to reduce the risk for coronary heart disease (CHD). The exact mechanism by which dietary PS consumption affect blood cholesterol level is not fully understood; however, it has been suggested that their greater hydrophobicity result in higher affinity for micelles than cholesterol [7]. Therefore, digested PS may displace intestinal cholesterol from the micelles, thus reducing intestinal cholesterol absorption and reabsorption [8]. Such a reduction results in compensatory increases in endogenous cholesterol synthesis [8] and higher LDL-receptor expression [9]. The net overall effect is that circulating LDL-cholesterol concentrations are lowered. However, PS intervention could also lead to a small reduction in plasma concentrations of lipophilic carotenoids including β-carotene as well as vitamin A and possibly vitamin E [10-12].

Accumulated evidence has associated dietary PS impact on circulating LDL-cholesterol with their solubilization in the provided preparations [see review in [13]]. In several reports, emulsification of PS with lecithin, sucrose esters, DAG or in different food matrices was suggested as increasing their bioavailability into the intestinal micelles [14]. Nonetheless, PS esterification to long-chain fatty acid (FA) is the most common solubilization method in food preparations [10,15] and dietary supplements [16,17]. Esterifying PS to vegetable oils FA from sunflower, soybean, or rapeseed increases their incorporation into fat-containing products by 10-fold while maintaining their cholesterol absorption inhibiting functions [10,15]. In contrast to the current practice in the food industry, recent studies used animal fat, such as beef tallow [18] or fish oil (FO) [19-22], as the FA source for PS esterification. Consumption of the latter was reported to favorably affect other key and independent risk factors associated with CHD, such as elevated plasma triglyceride levels [23]. Indeed, PS esters of fish-oil FA provided to animal models favorably altered both non-high density lipoprotein (HDL)-cholesterol and triglyceride serum concentrations [20-22].

Most of the randomized clinical trials testing food matrices containing PS in normo- to hyper-cholesterolemic individuals [10,15] have focus on their cholesterol lowering properties. Additionally, several studies have further examined the impact of these PS preparations on lipid peroxidation [24-26], endothelial markers [26,27], inflammation markers [16,26,28-30], as well as their anti-cancer effects [see review in [31]]. However, systematic investigation of the impact of different FA classes esterified to PS on plasma cholesterol concentrations or other risk factors has not yet been carried out in human. Our objective was to compare the effects of PS esterified to sunflower oil (omega-6; PS-SO), olive oil (omega-9; PS-OO), or FO (omega-3; PS-FO) FA on lipids profile, fat-soluble vitamins bioavailability, as well as on inflammation- and coagulation-related marker levels in mildly overweight, hyperlipidemic subjects.

## Results

### Subjects

The study was initiated with 11 males and 13 females who met the trial criteria. There were three dropouts due to personal reasons (n = 2) and FO-associated gastrointestinal discomfort (n = 1). Baseline characteristics of the 21 subjects who completed the study are shown in Table 1. Mean baseline body weight, as well as the indicated plasma lipids, did not differ between the PS containing treatments (data not shown).

**Table 1:** Baseline characteristics of the study subjects (*n *= 11 males, 10 females)

Age (y)	54.19 ± 1.62^1^
Weight (kg)	73.69 ± 2.72
BMI (kg/m^2^)	25.93 ± 0.62
Total cholesterol (mmol/L)	6.09 ± 0.18
LDL-cholesterol (mmol/L)	3.91 ± 0.12
HDL-cholesterol (mmol/L)	1.28 ± 0.07
Triacylglycerol (mmol/L)	1.77 ± 0.25

^1 ^Values are means ± SEs.

### Plasma lipid levels

Plasma lipid concentrations at the end of the different PS ester treatments are shown in Table 2. No differences were detected in total cholesterol or LDL-cholesterol concentrations following supplementation of OO-based diet with PS esterified to SO, OO, or FO FA (Table 2). Nonetheless, consumption of these dietary matrices, i.e. PS-SO, PS-OO, or PS-FO significantly lowered total cholesterol (an effect size of -0.61, -0.66, or -0.72, respectively), and LDL-cholesterol (an effect size of -0.58, -0.55, or -0.45, respectively) concentrations, when compared to the indicated phases baseline values (data not shown). A tendency towards a reduction (Table 2) in HDL-cholesterol concentrations was also observed, albeit the overall impact was fairly small. Effects of these PS containing supplements on fasting and postprandial triglycerides plasma concentrations differed significantly (Table 2). PS-FO resulted in lower fasting triglyceride concentrations than those observed with PS-SO (*P *= 0.0001) and PS-OO (*P *< 0.0001). Similar differences between these interventions were shown in postprandial triglyceride concentrations (*P *< 0.0001). Consumption of PS esterified to vegetable oil FA, i.e. PS-SO and PS-OO, had comparable (*P *= 0.7404) and relatively small (an effect size of -0.22, and -0.25, respectively) impacts on fasting and postprandial (*P *= 0.8380) triglyceride concentrations.

**Table 2:** Endpoint fasting and postprandial plasma lipid concentrations (n = 21)

	PS-SO^2^	PS-OO	PS-FO	*P *value^3^
Cholesterol				
Total (mmol/L)	5.61 ± 0.21^1^	5.49 ± 0.25	5.48 ± 0.23	0.5793
LDL (mmol/L)	3.59 ± 0.16	3.48 ± 0.19	3.73 ± 0.17	0.2704
HDL (mmol/L)	1.29 ± 0.07	1.23 ± 0.07	1.30 ± 0.07	0.0767^†^
Triacylglycerols				
Fasting (mmol/L)	1.62 ± 0.19^a^	1.75 ± 0.25^a^	0.99 ± 0.12^b^	< 0.0001
Postprandial (mmol/L)	2.56 ± 0.23^a^	2.70 ± 0.23^a^	1.53 ± 0.15^b^	< 0.0001

^1 ^Values are means ± SEs.

^2 ^PS-SO: plant sterols esterified with sunflower oil fatty acids; PS-OO: plant sterols esterified with olive oil fatty acids; PS-FO: plant sterols esterified with fish oil fatty acids

^3 ^*P *values obtained by repeated-measures ANOVA with ^† ^baseline concentrations included in the model as covariates. Values of plasma triglyceride concentrations were normalized using a log transformation.

^*a, b *^Values not sharing a common superscript letter are significantly different at *P *< 0.05.

### Fat-soluble vitamins

Consumption of the various PS containing preparations had different effects on plasma concentrations of retinol and β-carotene, but not α-tocopherol (Table 3). Specifically, the PS-FO-fed group exhibited higher retinol concentrations as compared with the PS-OO (*P *= 0.0425) but not the PS-SO (*P *= 0.0638) groups. In addition, plasma β-carotene concentrations following PS-FO feeding were elevated in comparison with PS-SO (*P *= 0.0139), but not PS-OO (*P *= 0.2376). No differences between PS-SO and PS-OO groups were detected in retinol and β-carotene concentrations (*P *= 0.9974, and 0.5302), respectively.

**Table 3:** Endpoint serum fat-soluble vitamin concentrations (n = 21)

	PS-SO^2^	PS-OO	PS-FO	*P *value^3^
Retinol (μg/dL)	65.0 ± 2.3^1, ab^	64.7 ± 2.1^a^	70.2 ± 2.3^b^	0.0244
β-carotenes (μg/dL)	36.2 ± 3.4^a^	35.8 ± 3.3^ab^	42.0 ± 4.5^b^	0.0169
α-tocopherol (μg/dL)	1773 ± 138	1901 ± 117	1764 ± 142	0.2108

^1^Values are mean ± SEs.

^2 ^PS-SO: plant sterols esterified with sunflower oil fatty acids; PS-OO: plant sterols esterified with olive oil fatty acids; PS-FO: plant sterols esterified with fish oil fatty acids

^3 ^*P *values obtained by repeated-measures ANOVA.

^*a, b *^Values not sharing a common superscript letter are significantly different at *P *< 0.05.

### Circulating inflammatory markers and blood coagulation markers

Effects of different PS esters containing treatments on plasma inflammatory and coagulation markers are shown in Table 4. Plasma tumor necrosis factor – α (TNF-α), interleukin-6 (IL-6), C-reactive protein (CRP), prostate specific antigen (PSA), and fibrinogen concentrations were unaffected by the consumption of the different dietary treatments (Table 4). However, the consumption of PS-FO resulted in lowered plasminogen activator inhibitor 1 (PAI-1) concentrations compared with PS-SO (*P *= 0.0282), but not PS-OO (*P *= 0.7487). There were no differences (*P *= 0.2080) between the effects of PS esterified to SO and OO FA on PAI-1 concentrations.

**Table 4:** Endpoint plasma inflammation- and coagulation-related markers (n = 21)

	PS-SO^2^	PS-OO	PS-FO	*P *value^3^
Inflammation				
TNF-α (ng/L)	1.03 ± 0.19^1^	1.03 ± 0.21	1.06 ± 0.24	0.9046
IL-6 (ng/L)	1.77 ± 0.22	1.91 ± 0.22	1.50 ± 0.17	0.1501
CRP (mg/L)	1.42 ± 0.48	1.48 ± 0.49	1.71 ± 0.63	0.8656
PSA^4 ^(μg/L)	0.92 ± 0.10	0.84 ± 0.08	0.89 ± 0.09	0.6102
Coagulation				
PAI-1 (μg/L)	40.3 ± 11.5^a^	26.6 ± 5.5^ab^	20.5 ± 2.6^b^	0.0297
Fibrinogen (g/L)	3.41 ± 0.15	3.42 ± 0.11	3.25 ± 0.11	0.2217

^1 ^Values are means ± SE's.

^2 ^CRP: C-reactive protein; IL-6: Interleukin-6; PAI-1: Plasminogen Activator Inhibitor 1; PSA: prostate specific antigen; PS-SO: plant sterols esterified with sunflower oil fatty acids; PS-OO: plant sterols esterified with olive oil fatty acids; PS-FO: plant sterols esterified with fish oil fatty acids; TNF-α: Tumor necrosis factor – α

^3 ^*P *values obtained by repeated-measures ANOVA.

^*a, b *^Values not sharing a common superscript letter are significantly different at *P *< 0.05.

^4 ^Chronic inflammation was suggested to play a role in the pathogenesis and progression of benign prostatic hyperplasia [54], which is associated with elevated PSA levels; n = 10

## Discussion

The present findings show that in healthy mildly overweight hypercholesterolemic subjects consumption of PS-FO differs from PS-OO and PS-SO in its impact on plasma triglyceride and fat-soluble vitamin levels. In addition, the PS-FO treatment resulted in lowered PAI-1 concentrations as compared to the interventions with PS-SO, but not PS-OO. Taken together, these observations suggest that the PS carriers may play, at least in part, a role in the exerted effects of various PS-preparations.

To date, PS esters provided as food matrices, mostly as spreads or in soft gel capsules, have been shown to affect LDL-cholesterol concentrations in both normo- and hypercholesterolemic individuals [10,15-17,32] However, the influence of the FA esterified to PS on the overall PS ester effect remains poorly studied. We have compared the cholesterol-lowering abilities of PS esterified to different FA. No differences between these treatments on blood cholesterol were detected. It has been previously shown that consumption of commercial PS ester spreads, such as PS-SO spread, has an impact on LDL-cholesterol concentrations comparable to that of PS-FO [19,28] or PS-OO [25]. Preliminary observations have linked the type of food matrix enriched with PS esters to different cholesterol-lowering effects [33]. In contrast, PS esterified to stearic acid were recently shown to be more effective than PS-SO in lowering both blood and liver cholesterol concentrations in hamsters [18]. The exact mechanism by which these supplements elicit differential effects on blood cholesterol concentrations remained unclear; nevertheless, the authors speculated that these findings may be associated with the combined effects of free PS and stearic acid. These observations may suggest that the cholesterol-lowering properties of PS esters of long-chain unsaturated FA differ from that of long-chain saturated FA.

In the current study, the PS-FO-fed group exhibited lowered fasting and postprandial triglyceride concentrations beyond that observed with PS-SO and PS-OO. The hypotriglyceridemic effect of omega-3 FA consumption is well-established [34]. Recently, we reported that PS-FO administration results in a greater reduction in triglyceride concentrations in comparison to FO consumption [19]. In recent reports, vegetable oil FA esters of plant stanols were shown to lower triglyceride concentrations in metabolic syndrome subjects [35]. This effect was associated with decreased very-low density lipoprotein production rates and apolipoprotein CII and apolipoprotein CIII concentrations. These findings suggest that the obtained PS-FO triglyceride-lowering effect may involve multiple mechanisms of both free PS and omega-3 FA. Elucidating the exact metabolic pathway by which PS-FO affect circulating lipids warrants further study.

In addition to the reduction in the absorption of cholesterol, daily consumption of PS esters for several weeks effectively impedes the absorption in the gut of the lipophilic carotenoids, such as β-carotene, lycopene and α-tocopherol [10-12]. However, it was reported that the consumption of a controlled diet [36], or increasing dietary carotenoid intake [37], may minimize this effect. In our study, consumption of PS esters of vegetable oil as part of an OO-based diet affected the plasma β-carotene and retinol concentrations to a different degree as did PS esters of omega-3 FA (PS-FO). Nonetheless, no differences were detected between the PS-SO and PS-OO groups. Dietary β-carotene absorption in the intestine was shown to be mediated via its solubilization in mixed micelles with bile components, lecithin and hydrolyzates of dietary lipids [38]. It was previously speculated that depletion of carotenoids from these micelles in the presence of PS was dependent on the PS dosage, the background diet, and the vehicle in which PS were solubilized [13]. When spreads containing sitostanol esters of soybean, sheanut, or rice bran oil FA were compared, all reduced lipid-standardized carotenoids albeit to a variable extent (9–43%) [39]. It has been recently reported [12] that long-term consumption of PS esters decreased plasma β-carotene concentrations more than did free PS. In the PS-FO treatment the proportion of PS-esters to free PS (~22%:5%) was somewhat lower in compare to the PS-SO and PS-OO (~28%:1.5%) supplements, but it is unlikely that the obtained results in such a short intervention are associated with these differences. Furthermore, enhanced β-carotene bioavailability and its conversion into vitamin A were reported in animals consuming micelles enriched with omega-9 and omega-3 rather than omega-6 FA [40]. Our findings, as well as those of others, suggest that solubilization of dietary carotenoids in mixed micelles is affected by the PS vehicle, and that its characteristics contribute, at least in part, to their bioavailability.

Several reports are favoring PS consumption for additional properties aside from their cholesterol-lowering characteristics, including anti-oxidation, anti-inflammation, and anti-cancer [25,31,41,42] effects. The impact of PS containing matrices on key risk factors for CHD, such as CRP and other inflammation markers, is still controversial [16,26,28-30]. Conversely, accumulated *in vitro *data suggest that β-sitosterol may affect prostate cancer development [43-46]. In our study, supplementation of PS esters containing different FA did not affect plasma concentrations of inflammatory and coagulation markers, but did alter PAI-1 levels. Previous observations showed that consumption of PS esters [26,27] or FO [47] or both [48] had no impact on the endothelial function marker PAI-1. One possibility to explain this discrepancy might be the presence of DAG in both PS-FO and PS-OO treatments (1.4 g/d; 7:2 1,3:1,2-DAG) in the current study. Indeed, 3-month DAG ingestion by Japanese patients with type 2 diabetes resulted in lowered PAI-1 concentrations compared with the triacylglycerol oil group [49]. Though the dosage of DAG in the present study was considerably lower than in other clinical investigations [50], its consumption might have contributed to the observed action on PAI-1 concentrations.

## Conclusion

Present data show that the effects of PS esters of omega-3 FA on circulating lipids, hydrocarbon carotenoids bioavailability and PAI-1 concentrations differ from those of PS-SO and PS-OO in mildly overweight, hypercholesterolemic subjects. These findings may suggest that the FA moiety of PS esters and the other preparation components, such as DAG, may provide an additional contribution to the overall beneficial impact. Therefore, in the context of a Mediterranean-like diet, consumption of PS-FO could exert in hyperlipidemic subjects a more comprehensive lipid lowering effect than vegetable oil esters of PS while reducing the risk of decreases in plasma carotenoid concentrations.

## Methods

### Subjects

The study protocol and the informed consent were reviewed and approved by the Human Ethical Review Committee of the Faculty of Agriculture and Environmental Sciences for the School of Dietetics and Human Nutrition at McGill University (protocol number REB# 808-0403). All subjects received explanations about the protocol and signed consent forms were obtained from each participant.

Subjects were recruited by newspaper advertisement in Montreal, Canada, and the surrounding areas. Potential candidates were screened according to the following criteria at Mary Emily Clinical Nutrition Research Unit of McGill University. Inclusion criteria included men and women, baseline LDL-cholesterol 2.6 mmol/l (100 mg/dl), BMI ranging from 24 to30 kg/m^2 ^and aged 30–65 years. Candidates were excluded from the study if they had taken medications known to affect lipid metabolism, such as cholestyramine, colestipol, gemfibrozil, probucol, HMG-CoA reductase inhibitors; consumed FO capsules and/or supplements containing PS during the previous 3 months; diagnosed with diabetes mellitus, kidney disease or liver disease; were smokers; consumed more than two glasses per day of alcoholic beverages; or took two or more doses per week of laxatives or concentrated sources of fibres.

Power calculation, based on a 0.56 mmol/l (standard deviation of 0.84 mmol/l) change in the primary outcome, LDL-cholesterol levels [51], at the 0.05 level of significance and 80% power suggested that at least twenty subjects would be required. Twenty-four volunteers, eleven males, thirteen postmenopausal females, were recruited according to the study protocol. Table 1 summarizes the baseline characteristics of the twenty-one participants that completed the study.

### PS preparations

The low-fat PS-SO margarine (Take control^®^) was kindly donated by Unilever Bestfoods NA (Baltimore, MD). Both the PS-OO and PS-FO oil supplements were manufactured by Enzymotec LTD (Migdal HaEmeq, Israel). These PS esters matrices were synthesized by a novel process in which PS were esterified to OO- or FO-FA while producing DAG [52]. Table 5 lists the composition of the different treatments. The FA composition of the PS-matrices were identified according to American Oil Chemist' Society official method and recommended practice Ce 1–62 (Champaign, IL). Briefly, the FA profiles were analyzed by methanolysis of the FA in a sample of margarine or oils extracted with a methanolic NaOH solution using BF3 as catalyst. The methyl esters were extracted with n-heptane and analyzed with a gas chromatograph (Agilent; Agilent Technologies, Palo Alto, CA) equipped with CP-Sil 88 column (Supelco, Bellefonte, PA) and a flame ionization detector on the basis of comparison of retention times equal to those of authentic standards. All PS supplementations: 21.4 g low-fat PS-SO margarine, 9.1 g PS-OO oil, and 9.6 g PS-FO oil provided an equivalent of 1.7 g soyabean sterols per day. PS-OO and PS-FO contained 1.4 g/d DAG (7:2 of 1,3:1,2-DAG) as well, and PS-FO provided a total of 1.7 g/d eicosapentaenoic acid and 3.7 g/d docosahexaenoic acid. Tocopherol mixtures (0.2 % (*w*/*w*)) were added to the base stock FO used as a source of FA for the esterification of PS in the PS-FO preparation. No further antioxidant beyond the original formulation was introduced to the tested PS-FO or to other PS-matrices.

**Table 5:** Fatty acid and plant sterol composition of study formulations

	PS-SO^2^	PS-OO	PS-FO
Plant sterol esters (g/100 g of oil or margarine)	12.1^1^	28.6	22.1
Free plant sterol equivalents (g/100 g of oil or margarine)			
β-sitosterol	3.7	8.4	8.1
Campesterol	1.9	4.6	4.9
Stigmasterol	1.4	3.9	3.3
Brassicasterol	0.2	0.5	0.8
Others	0.5	1.0	1.1
Total	7.8	18.3	18.2
Fatty acids (% by weight of total fatty acids)			
12:0	0.2	ND	ND
14:0	0.2	ND	0. 5
16:0	8.3	12.8	1.1
16:1	0.1	0.9	0.7
17:0	0.1	ND	0.2
18:0	6.2	3.0	0.5
18:1	41.8	70.6	7.5
18:2	36.4	10.7	1.2
18:3 n-3	5.5	0.8	1.3
18:4	ND	ND	4.0
20:0	0.5	0.5	0.1
20:1	0.3	0.2	3.1
20:4 n-6	ND	ND	0.7
20:4 n-3	ND	ND	2.6
20:5 n-3	ND	ND	14.8
22:0	0.4	0.2	ND
22:1	ND	ND	0.7
22:5 n-3	ND	ND	4.6
22:6 n-3	ND	ND	49.0
24:0	0.2	0.1	0.6
24:1	ND	ND	0.2
Other fatty acids	ND	0.1	0.6

^1 ^Typical values; provided by Unilever and analyzed as previously described [39].

^2 ^PS-SO: plant sterols esterified with sunflower oil fatty acids; PS-OO: plant sterols esterified with olive oil fatty acids; PS-FO: plant sterols esterified with fish oil fatty acids; ND, not detected.

### Experimental design

The study was a semi-randomized, crossover, double blind clinical trial using a Latin square sequence. It consisted of three phases of 28 d separated by a 4-week washout interval. During each supplementation phase, the subjects were provided by the clinic with an OO-based, weight-maintaining, North-American diet, and throughout the washout period, they consumed their own habitual diets. This OO-based diet contained approximately 15% energy as protein, 55% energy as carbohydrates, 30% energy as fat, of which approximately 70% was provided by OO, 80 mg cholesterol per 1000 kcal, and 12 g fibre per 1000 kcal. Three isoenergetic meals (breakfast, lunch and supper) were prepared according to a 3-d cycle menu in the metabolic kitchen of the clinic, where the foods were weighed precisely to 0.5 g during meal preparation. Supplements were ingested at breakfast under supervision to monitor compliance. The subjects were instructed to consume only foods and beverages provided by the clinic, and were encouraged to keep a constant exercise level throughout the study to ensure that bodyweights remained unchanged.

Fasting blood samples were collected from the subjects on days 1, 2, 28 and 29 of each phase. On the 28^th ^day of each phase, plasma postprandial triglyceride concentrations were measured 4 h after breakfast.

### Plasma lipid analysis

Serum total cholesterol, HDL-cholesterol, and triglyceride concentrations were measured by automated methods on the multianalyzer Dimension RxL Max utilizing enzymatic reagents Flex (Dade Behring Diagnostic, Marburg, Germany). LDL-cholesterol concentrations were calculated using the Friedewald equation, unless the triglyceride concentrations were > 4.5 mmol/l, in which case the LDL- cholesterol concentrations were measured directly.

### Plasma inflammation and coagulation markers

The serum TNF-α (Quantikine HS, R&D Systems Inc., Minneapolis, MN) and IL-6 (Quantikine HS, R&D Systems Inc.), and the plasma CRP (Roche Diagnostics Co., Indianapolis, IN), PSA (Abbott IMX, Montreal, Quebec, Canada), and PAI-1 (PAI-1 Asserachrom, Diagnostica Stago, Asnieres sur Seine, France) concentrations were determined using the respective enzyme immunoassay kits, according to the manufacturer's instructions. Fibrinogen levels were measured on citrated plasma aliquot by automated clot-rate assay based on the original method of Clauss using the ACLAdvance instrument (Beckman Coulter, Fullerton, CA).

### Plasma fat-soluble vitamin concentrations

Plasma concentrations of the fat-soluble vitamins, retinol, α-tocopherol, and β-carotene were measured by using light-protected, reverse-phase HPLC as described elsewhere [19,53]. Briefly, the fat-soluble components were extracted from plasma using methanol and hexane (1:5 *v*:*v*) in the presence of internal standards (Sigma-Aldrich, St Louis, MO); retinol acetate for retinol and tocopherol acetate for α-tocopherol and β-carotene. The organic layer was then transferred, dried, dissolved in methanol, and injected into an HPLC column (JASCO, Dunmow, United Kingdom) equipped with an ultraviolet detector and an auto-injector system. The fat-soluble vitamins were identified and quantified using authentic standards (Sigma-Aldrich), the corresponding standard curves, and multi-wavelength detection.

### Statistical analyses

All data were expressed as means and their standard errors (SE). Statistical significance was set at α = 0.05 for all analyses. Differences in plasma variables were tested by repeated-measures ANOVA with the type of dietary matrix in each intervention arm as the within-subject factor and with endpoint values as the dependent variable. Baseline values were inserted into the model as covariates if their interaction with dietary matrices was found to be statistically significant. Subsequently, contrast analyses were used to identify differences between pairs of diets. Furthermore, a modified Cohen's effect size was calculated for plasma lipid endpoint values to evaluate changes from the baseline values. Data were analyzed using SAS software (version 8.0; SAS Institute Inc, Cary, NC, USA).

## Abbreviations

CHD = Coronary heart disease

CRP = C-reactive protein

DAG = Diacylglycerol

FA = Fatty acid

FO = Fish oil

HDL = High density lipoprotein

IL-6 = Interleukin-6

LDL = Low-density lipoprotein

OO = Olive oil

PAI-1 = Plasminogen activator inhibitor 1

PS = Plant sterols

PSA = Prostate specific antigen

PS-FO = Plant sterols esterified to fish oil fatty acids

PS-OO = Plant sterols esterified to olive oil fatty acids

PS-SO = Plant sterols esterified to sunflower oil fatty acids

SE = Standard error

SO = Sunflower oil

TNF-α = Tumor necrosis factor – α

## Competing interests

DP and YH are Director of Clinical Studies and Project Manager at Enzymotec LTD, respectively; PJHJ, ID, and YMC have no competing interests. ID was partially supported by a Post-Doctoral Industrial Research Fellowship from the Natural Sciences and Engineering Research Council of Canada. This study was funded by Enzymotec LTD, Israel.

## Authors' contributions

Peter J. H. Jones, Ph.D. designed the study. Isabelle Demonty, Ph.D. acted as the study coordinator. Yen-Ming Chan provided assistance for study coordination. Yael Herzog, Ph.D. was responsible for the fat-soluble vitamin analyses. Dori Pelled, Ph.D. was responsible for the statistical analyses. All authors participated in data interpretation, and manuscript preparation. All authors have read and approved this manuscript.
